# Maritime workers and their global health: Need to improve scientific knowledge and prevention

**DOI:** 10.7189/jogh.12.03031

**Published:** 2022-05-23

**Authors:** David Lucas, Victorita Corman, Olaf C Jensen, Ilona Denisenko, Don E-III Lucero-Prisno, Maria L Canals

**Affiliations:** 1 ORPHY Laboratory, University Brest, Brest, France; 2 Occupational and Environmental Diseases Center, Teaching Hospital, Brest, France; 3 French Society of Maritime Medicine Brest, Brest, France; 4 Centre for Maritime Health and Society, Department of Public Health, University of Southern Denmark, Esbjerg, Denmark; 5 Universidad Metropolitana de Educación Ciencia y Tecnología. Facultad de las Ciencias y Tecnología, Panamá, Republic of Panamá; 6 Regional Medical Office German Embassy, Moscow, Russia; 7 London School of Hygiene and Tropical Medicine, London, UK; 8 University of the Philippines Open University, Los Baños, Laguna, Philippines; 9 University of Cadiz FUECA, Cadiz, Spain; 10 Sociedad Española de Medicina Marítima (SEMM) /Sanidad Marítima, Tarragona, Spain

The maritime industry is one of the most significant contributors to globalisation as this sector accounts for 90% of global trade. As shown by the SARS CoV-2 pandemic and the Ukrainian crisis, rapid geopolitical changes and national and international regulations have a strong impact on maritime workers’ occupational exposure patterns. In the maritime sector, most of the regulations, including fitness at work and safety, are governed by international organisations. Very few sectors can claim such a dialogue on a global scale. Despite this, there is very little monitoring of the occupational environment for the workers at sea and only a few high impact epidemiological studies have been published in this field. There is a real need for new scientific knowledge on occupational exposures and the health of maritime workers to improve information and training on risk management in this population.

The Maritime Health Research and Education-NET (MAHRE-Net) is a non-profit network whose primary aim is to provide a network for an enhanced evidence base for the identification of health risks and gains related to occupation and employment to foster safe and healthy preventive strategies and policies.

The maritime industry is one of the most significant contributors to the process of globalisation as this sector accounts for 90% of global trade [1]. Even in 2012, 38 000 students were enrolled in a maritime academy in UE and Norway. In an Oxford economics study, it was estimated that the maritime cluster (ports, shipping, maritime business services) supported £22 billion in GDP and 489 000 jobs in the UK economy in 2013 [2].

As shown by the SARS CoV-2 pandemic and the current Ukrainian crisis, rapid geopolitical changes and changing patterns related to the climate, the economy and national and international regulations have a strong impact on the occupational exposure patterns of maritime workers [3-5].

**Figure:**
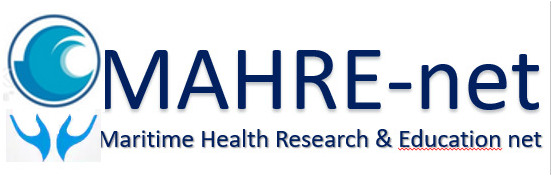
Photo: MahreNet Logo edited by MarheNetwork (used with permission).

Recently, the World Health Organisation (WHO) declared seafarers as essential workers and recommended they have priority access to COVID-19 vaccination [6].

Moreover, maritime workers have a high rate of occupational accidents. European statistics of occupational accidents show that the transportation, agricultural, forestry, and fishing industries have a high rate of accidents and especially fatal accidents [7]. Over one in five (21.0%) fatal accidents at work in the EU-28 in 2017 took place within the construction sector, closely followed by transportation (including road, rail, air and maritime transport) and storage (16.5%).Agriculture/forestry/fishing (13.2%) was the only other economic section to record a double-digit share of the total number of fatal accidents [8]. The number of reported shipping casualties or incidents in the world actually increased by 5% to 2 815 in 2019 [8]. Most of them are linked to human factors and work performance [9]. Moreover, depression, boredom and fatigue have been described as risk factors [9-11].

In entire maritime sector (transportation, fishing, offshore, port) most regulations are developed by international organisations such as the International Maritime Organisation (IMO), International Labour Organisation (ILO), or the Food and Agriculture organisation (FAO) [12]. These organisations act on occurrences of new environmental and occupational risks such as fumigation of foodstuffs and shipping’s impact on air quality [13]. ILO published Guidelines on the medical examinations of seafarers to assist medical practitioners, shipowners, seafarers’ representatives, seafarers and other relevant persons with the conduct of medical fitness examinations of serving seafarers and seafarer candidates[14]. Health survey and medical certificate for seafarers are also under international regulations with the Convention on Standards of Training, Certification and Watchkeeping for Seafarers (STCW 78/95/2010 last amended called Manila Convention). This is a common and exemplary way of thinking leading to the implementation of shared standards worldwide. Very few sectors can claim such a dialogue at the world scale. But it should go further, to maximize the value of training centres in terms of skills and network [15,16].

However, the monitoring of the work environment that is routinely carried out for employees in land-based industries is far superior compared to that for workers at sea. For the last forty years, the National Institutes of Occupational Health and Safety in European countries have collected and analysed information on the workers' environment and health using questionnaires, but seafarers, fishermen, dockworkers, and offshore workers are not represented in these surveys [17]. Maritime industry workers make a significant contribution to the global economy, but studies show that their health received comparatively little attention [18].

Similarly, when looking at scientific literature, we found a 2012 review and position paper by McLachlan et al [18] which include 198 articles, with 13 to 35 articles published per year . In an extended search of the PubMed database with the MeSH Term “Naval Medicine” and a time range from 2010 to 2021, we found 666 articles. Indeed, by restricting the range to 2016-2021, 246 of 265 included articles were in the field of Maritime Health. Most of them were observational studies (32%), followed by position papers or letters to the editor (23%), case-reports (13%) and reviews (12%). Only 4 papers on cohort and 4 on case-control studies have been published during this time.

Studying adverse health and social outcomes related to exposures in the working environment or work tasks and working conditions is a major occupational and societal challenge. Results in terms of occupational and public health benefits may range from effective preventive measures to detection of possible health outcomes.

Indeed, training and education on safety and risk assessment for workers and health care workers involved in maritime world is a primary need [19]. General risk perception for communicable diseases and hygiene are low in maritime students. Among 23 themes identified to improve training, the following three domains were ranked the highest among maritime professionals: health safety and risk (83%), policy/rules/regulations (73%), and maritime health services (72%) [20].

There is a real need for new scientific knowledge on occupational exposures and the health of maritime workers to updated data for improving training on risk management in this population.

In this regard, we recently created a scientific core group. Our aim is to provide a network for an enhanced evidence base for the identification of occupation- and employment-related health risks and gains related to foster safe and healthy preventive strategies and policies. The Maritime Health Research and Education-NET (MAHRE-Net) [21] is a non-profit network of researchers, seafarers, fishers, other workers, and students composed of four main parts:

1. Educational programs on health science research methods [22].
2. Studies based on occupational health questionnaires in ergonomics, mental health, wellbeing, safety climate, and other standardised questionnaires.
3. Use of clinical health examination data for early diagnosis and prevention of Type 2 Diabetes Mellitus and Hypertension as important and preventive parts of the metabolic syndrome [23].
4. Systematic literature reviews.

We aim to provide a foundation for the evidence base to foster safe and healthy preventive strategies and policies within the UN Global Sustainable Goals, especially Goal 3: Good health and well-being for all workers and Goal 8: Decent Work and Economic Growth. Legal, social, gender equity, training, education, and prevention issues will be discussed in different interlinked working groups [21,24]. The action will foster safe and healthy preventive strategies and policies to advance ongoing long-term studies in this field. Three key methods were defined for achieving this aim: Improving and building of Cohort studies, creating Exposure assessment and Prevention measures, improving Equity, Health, and Education.
